# Supplementary material for: Wearable-derived cardiovascular fitness age and its lifestyle correlates in 442 adults
Source: Front Digit Health. 2026 Jul 7;8:1842633. doi: 10.3389/fdgth.2026.1842633 (PMC13385220; doi:10.3389/fdgth.2026.1842633)
Supplement: Supplementary file 1 [file Datasheet1.pdf]

Supplementary Material

1 SUPPLEMENTARY TABLES AND FIGURES

Table S1: Consolidated variable definitions and within-Ultrahuman validation references for all variables used in this study. ‡ marks direct algorithm inputs to Cardio Age; † marks indirect constituents (variables that share algorithmic components with Cardio Age or are known physiological determinants of a direct input). Variables without either symbol have no direct or indirect algorithmic link to Cardio Age, except for self-reported demographics. Composite scores are described by their input signals and intended interpretation only; weights, thresholds, and formulae are proprietary and not disclosed. PPG, photoplethysmography; NSDR, non-sleep deep rest.

Variable	Definition	Unit	Within-Ultrahuman validation
Nighttime resting heart rate‡	Mean heart rate during the nighttime sleep window, derived from PPG.	bpm	Validated against Apple Watch and FDA-approved SleepImage: MAE 2.4–2.6 bpm; session-level Spearman $\rho \approx 0.99$ (Krishnan et al., 2024).
HRV (RMSSD)‡	Heart rate variability, root mean square of successive RR-interval differences, during nighttime sleep.	ms	Present analyses rely on within-person HRV trends, which consumer-wearable literature supports for monitoring physiological adaptation (Plews et al., 2013), rather than absolute values (see Manuscript Section §3).
Estimated VO_2 max‡	Predicted maximal oxygen uptake from a proprietary weighted combination of established CRF estimation methods (Tanaka et al., 2001; Jackson et al., 2009) with nighttime RHR as a foundational input.	mL/kg/min	Derived from established CRF estimation methods; present analyses rely on within-person longitudinal trajectories rather than absolute values (see Manuscript Section §3). External validation against laboratory cardiopulmonary exercise testing is the subject of a separate study in preparation.
Recovery Score†	Proprietary daily 0–100 score from nighttime RHR, HRV, skin temperature, and Stress Rhythm Score, with additive contributions from breathwork, NSDR, and nap sessions.	0–100	Platform composite; no external validation.
Stress Rhythm Score†	Proprietary daily 0–100 score from nighttime RHR, HRV, and the daily pattern of heart-rate excursions above resting baseline contextualized by circadian phase.	0–100	Platform composite; no external validation.
BMI†	Body mass index, computed from self-reported height and weight.	kg/m^2	Standard anthropometric calculation; self-reported inputs.
Weight†	Self-reported body weight.	kg	Self-reported.
Sleep duration (total sleep time)	Total time spent in sleep across the nighttime sleep window.	min	Validated against Somfit single-channel EEG system over 20 paired nights: $r = 0.889$, ICC $= 0.844$, bias $+1.0$ min (Driller et al., 2026).
Sleep onset time	Clock time of sleep onset, derived from PPG- and accelerometer-based sleep staging.	h:mm	Validated against Somfit: $r = 0.9998$, ICC $= 0.9997$, bias -1.4 min (Driller et al., 2026).
Sleep offset time	Clock time of sleep offset.	h:mm	Validated against Somfit: $r = 0.985$, ICC $= 0.974$, bias $+3.6$ min (Driller et al., 2026).
Sleep efficiency	Total sleep time divided by total time in bed, expressed as a percentage.	%	Present analyses rely on within-person trends (see Manuscript Section §3).

(Table S1 continued)

Variable	Definition	Unit	Within-Ultrahuman validation
Deep sleep duration	Time scored as slow-wave (N3) sleep within the nighttime sleep window.	min	Both deep and REM sleep durations are inputs to the Ultrahuman Sleep Score, characterized externally as a behaviorally meaningful composite at population scale (Dhawale et al., 2026) (see Manuscript Section §3).
REM sleep duration	Time scored as rapid-eye-movement sleep within the nighttime sleep window.	min	See Deep sleep row and Manuscript Section §3 for the Sleep Score composite framing.
Daily steps	Daily step count derived from accelerometer data.	steps	Accelerometer-derived; analyzed as a within-person lifestyle correlate.
Active hours	Waking hours with sufficient step activity, excluding sleep, naps, and non-wear; workout periods auto-classified as active.	hours/day	Platform-derived; no external validation.
Movement Index	Proprietary daily 0–100 score combining active hours, inactive waking time, daily steps relative to a personalized goal, and weekly workout frequency and volume.	0–100	Platform composite; no external validation.
Skin temperature (peripheral)	Peripheral skin temperature measured by the Ring AIR at 5-minute intervals; used as a Recovery Score input.	°C	Validated against the OvuSense SWS (CE Class IIa medical device): bias -0.114 °C, MAE 0.046 °C (Gupta et al., 2025). Also validated against the iButton DS1921H research reference under off-body and on-body conditions: Spearman $\rho = 0.965$, bias near zero, narrow limits of agreement (Shanmugam et al., 2025).
Age (chronological)	Self-reported chronological age at study entry.	years	Self-reported.
Sex / gender	Self-reported sex/gender (Female, Male, Other).	category	Self-reported.
Height	Self-reported standing height.	cm	Self-reported.
Self-reported mobility level	Onboarding categorical (Sedentary / Moderate / Active); influences the VO_2 max algorithm calibration as an adjustment factor.	category	Self-reported; algorithm adjustment factor only.

Table S2. All wearable-derived metrics in extreme Cardio Age gap groups (12-month window). Values are median (IQR). p-values from Mann-Whitney U tests. Metrics are grouped by type: constituent (‡, direct algorithm inputs), indirect constituent († , shares algorithmic components), and independent (no algorithmic link).

Metric	Type	Youngest Hearts	Oldest Hearts	Median Diff	p
RHR (bpm)‡	Constituent	47.9 (45.4, 49.4)	61.7 (59.2, 66.4)	−13.78	<0.001
HRV RMSSD (ms)‡	Constituent	55.9 (46.4, 68.9)	37.2 (32.1, 47.6)	+18.63	<0.001
VO_2 max (mL/kg/min)‡	Constituent	48.7 (45.3, 51.6)	36.4 (33.7, 38.6)	+12.32	<0.001
Recovery score†	Indirect constituent	74.2 (69.9, 76.9)	68.1 (63.1, 71.0)	+6.08	<0.001
Stress rhythm†	Indirect constituent	77.9 (73.3, 80.6)	77.5 (69.8, 81.8)	+0.38	0.759
BMI (kg/m^2)†	Indirect constituent	22.8 (21.0, 26.2)	29.6 (26.1, 33.3)	−6.78	<0.001
Weight (kg)†	Indirect constituent	69.0 (61.7, 77.7)	89.0 (83.6, 100.0)	−20.00	<0.001
Sleep duration (min)	Independent	454.6 (420.9, 474.0)	417.4 (390.2, 464.1)	+37.27	0.032
Sleep efficiency (%)	Independent	90.4 (88.3, 92.8)	88.6 (85.3, 90.7)	+1.83	0.002
Deep sleep (min)	Independent	73.1 (67.2, 80.7)	72.1 (66.0, 78.0)	+1.00	0.427
REM sleep (min)	Independent	120.5 (107.2, 133.0)	98.3 (90.4, 111.7)	+22.18	0.007
Daily steps	Independent	7221 (5171, 8816)	5540 (4074, 7727)	+1681	0.047
Active hours	Independent	8.4 (6.2, 9.8)	7.0 (5.9, 8.4)	+1.42	0.059
Movement index	Independent	70.8 (62.2, 76.5)	69.2 (56.7, 77.3)	+1.60	0.340

‡ Direct constituent (algorithm input). † Indirect constituent; shares algorithmic components.
Youngest Hearts: CA gap ≤ -5.4 years ($N = 45$); Oldest Hearts: CA gap $\geq +2.3$ years ($N = 45$). Sample sizes vary by metric due to data availability.

Table S3. Spearman rank correlations between Cardio Age gap and all wearable-derived metrics (12-month window). Bootstrapped 95% confidence intervals from 1,000 iterations.

Metric	Type	r	95% CI	N	p
RHR‡	Constituent	0.564	(0.497, 0.629)	442	<0.001
HRV‡	Constituent	−0.347	(−0.431, −0.258)	442	<0.001
VO_2 max‡	Constituent	−0.573	(−0.636, −0.500)	428	<0.001
Recovery score†	Indirect constituent	−0.320	(−0.406, −0.224)	442	<0.001
Stress rhythm†	Indirect constituent	−0.050	(−0.140, 0.039)	442	0.293
BMI†	Indirect constituent	0.278	(0.184, 0.365)	442	<0.001
Weight†	Indirect constituent	0.310	(0.227, 0.393)	442	<0.001
Sleep duration	Independent	−0.200	(−0.287, −0.097)	387	<0.001
Sleep efficiency	Independent	−0.194	(−0.285, −0.094)	387	<0.001
Deep sleep	Independent	−0.087	(−0.182, 0.016)	387	0.088
REM sleep	Independent	−0.203	(−0.290, −0.104)	387	<0.001
Steps	Independent	−0.145	(−0.240, −0.048)	430	0.003
Active hours	Independent	−0.123	(−0.212, −0.027)	430	0.011
Movement index	Independent	−0.076	(−0.165, 0.024)	432	0.116

‡ Direct constituent (algorithm input). † Indirect constituent; partly mediated through the algorithm.

Table S4. Body mass index and Cardio Age gap stratified by chronological age decade ($N = 442$). Mean BMI rose monotonically from the 18–29 decade through 50–59 then declined at 60+, where the sample is smallest ($N = 23$); Kruskal–Wallis across decades $H = 24.28$, $p < 0.001$. Mean Cardio Age gap followed a parallel pattern (Kruskal–Wallis $H = 20.71$, $p < 0.001$). Within-gender Spearman correlations between Cardio Age gap and chronological age were non-significant (Female $\rho = +0.035$, Male $\rho = +0.109$); the cohort-level correlation ($\rho = +0.238$) reflects gender composition.

Age group, y	N	Age, median (IQR)	BMI mean (SD), kg/m^2	BMI median (IQR)	CA gap mean (SD), y
18–29	192	25 (23, 27)	25.0 (5.6)	23.7 (21.4, 27.4)	−2.55 (2.45)
30–39	125	33 (31, 36)	26.2 (4.9)	25.2 (22.9, 29.1)	−1.55 (3.20)
40–49	66	45 (43, 47)	27.4 (6.1)	27.2 (23.0, 30.1)	−1.00 (3.27)
50–59	36	54 (53, 56)	28.9 (6.7)	27.1 (23.8, 32.9)	−0.88 (3.82)
60+	23	65 (62, 68)	25.7 (4.5)	25.4 (23.7, 27.9)	−1.52 (2.08)

Table S5. Twelve-month sustained trajectory profiles. Sustained improvers (delta < -2.0 years) and sustained worseners (delta $> +2.0$ years).

Characteristic	Sustained Improvers ($N = 52$)	Sustained Worseners ($N = 59$)
Mean age, years	33.1	35.4
Mean BMI, kg/m^2	26.4	26.8
Female, %	50.0	61.0
Mean delta CA, years	-3.24	$+3.49$

Table S6. Between-group longitudinal metric changes across the 12-month improver, stable, and worsener trajectory groups, classified by the direction of within-person Cardio Age change over the observation window. Group sizes vary by metric owing to data availability (e.g., resting heart rate $N = 148$ improvers / 100 stable / 178 worseners). p-values from Kruskal-Wallis tests.

Metric	Type	Improver Δ	Worsener Δ	p
RHR (bpm)‡	Constituent	-0.84	$+1.07$	<0.001
HRV RMSSD (ms)‡	Constituent	$+1.05$	-1.88	<0.001
VO_2 max (mL/kg/min)‡	Constituent	$+1.26$	-0.80	<0.001
Recovery score†	Indirect constituent	$+2.49$	$+1.52$	0.183
Sleep duration (min)	Independent	$+5.1$	-38.7	0.465
Sleep efficiency (%)	Independent	$+0.58$	-0.45	0.273
Deep sleep (min)	Independent	-4.04	-16.20	0.465
REM sleep (min)	Independent	-3.07	-22.91	0.361

Table S7. Multivariate linear regression of CA gap on independent predictors (12-month window). Primary model includes BMI†, sleep duration, sleep efficiency, gender, and age. Sensitivity model adds recovery score† to the primary model predictors. Standardized coefficients (β) reported.

Model	Predictor	β	p	N	Adj. R^2
Primary	BMI†	0.687	<0.001	379	0.301
	Sleep duration	0.063	0.657		
	Sleep efficiency	-0.409	0.005		
	Gender (female)	-1.377	<0.001		
	Age	-0.022	0.877		
Sensitivity	BMI†	0.497	<0.001	379	0.359
	Sleep duration	0.181	0.186		
	Sleep efficiency	-0.070	0.638		
	Recovery score†	-0.850	<0.001		
	Gender (female)	-1.382	<0.001		
	Age	0.109	0.430		

† Indirect constituent; partly mediated through the algorithm.

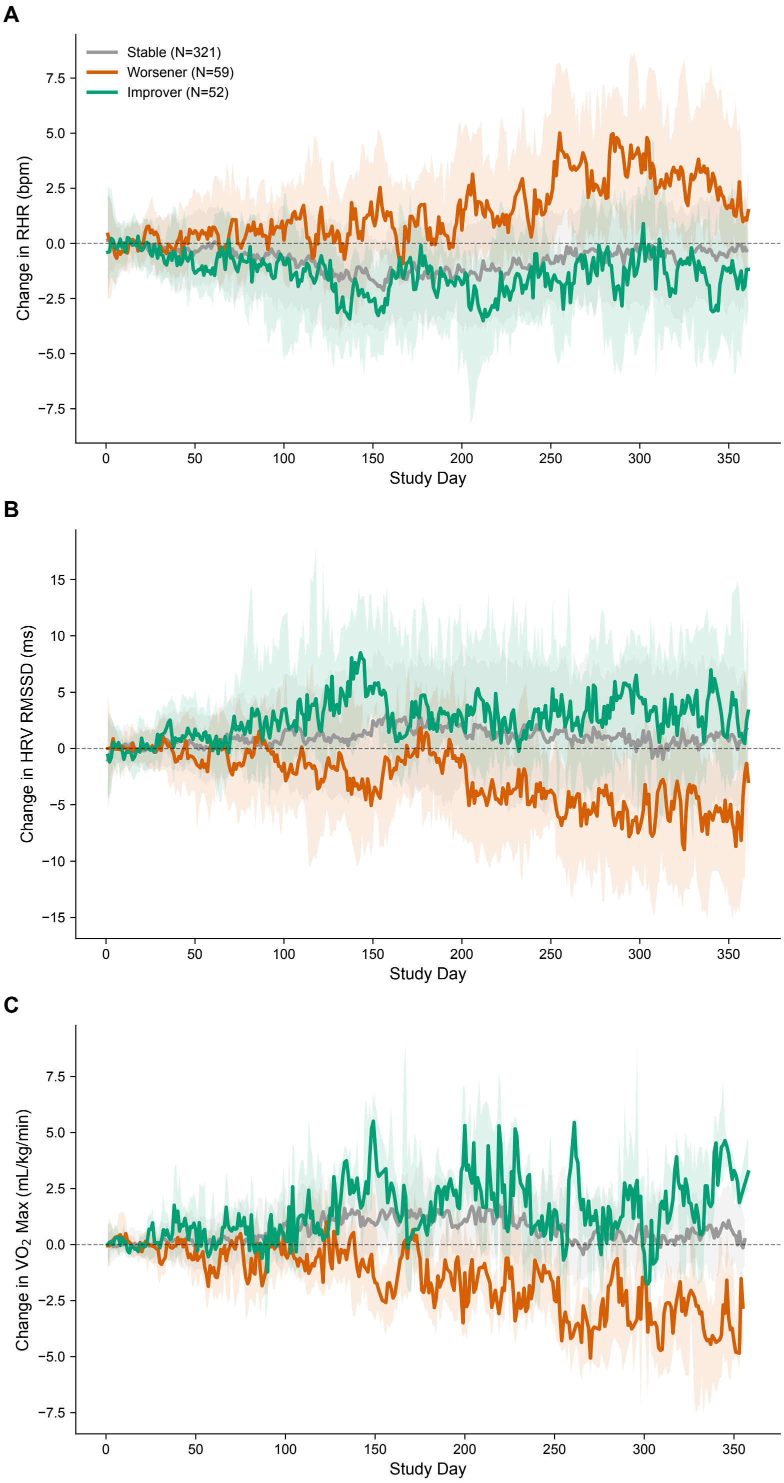

All three metrics are constituent inputs to the Cardio Age algorithm. Daily median ± IQR (7-day rolling, baseline = first 30-day mean).

Figure S1. Temporal divergence of constituent metrics between 12-month trajectory groups. Three panels show change from 30-day baseline in nighttime resting heart rate (top), HRV RMSSD (middle), and estimated VO_2 max (bottom) for sustained improvers ($N = 52$, green) and sustained worseners ($N = 59$, red). Divergence in resting heart rate and estimated VO_2 max becomes visible by months 3–4. All three metrics are direct constituent inputs to the Cardio Age algorithm; associations with Cardio Age trajectories are therefore partly tautological.

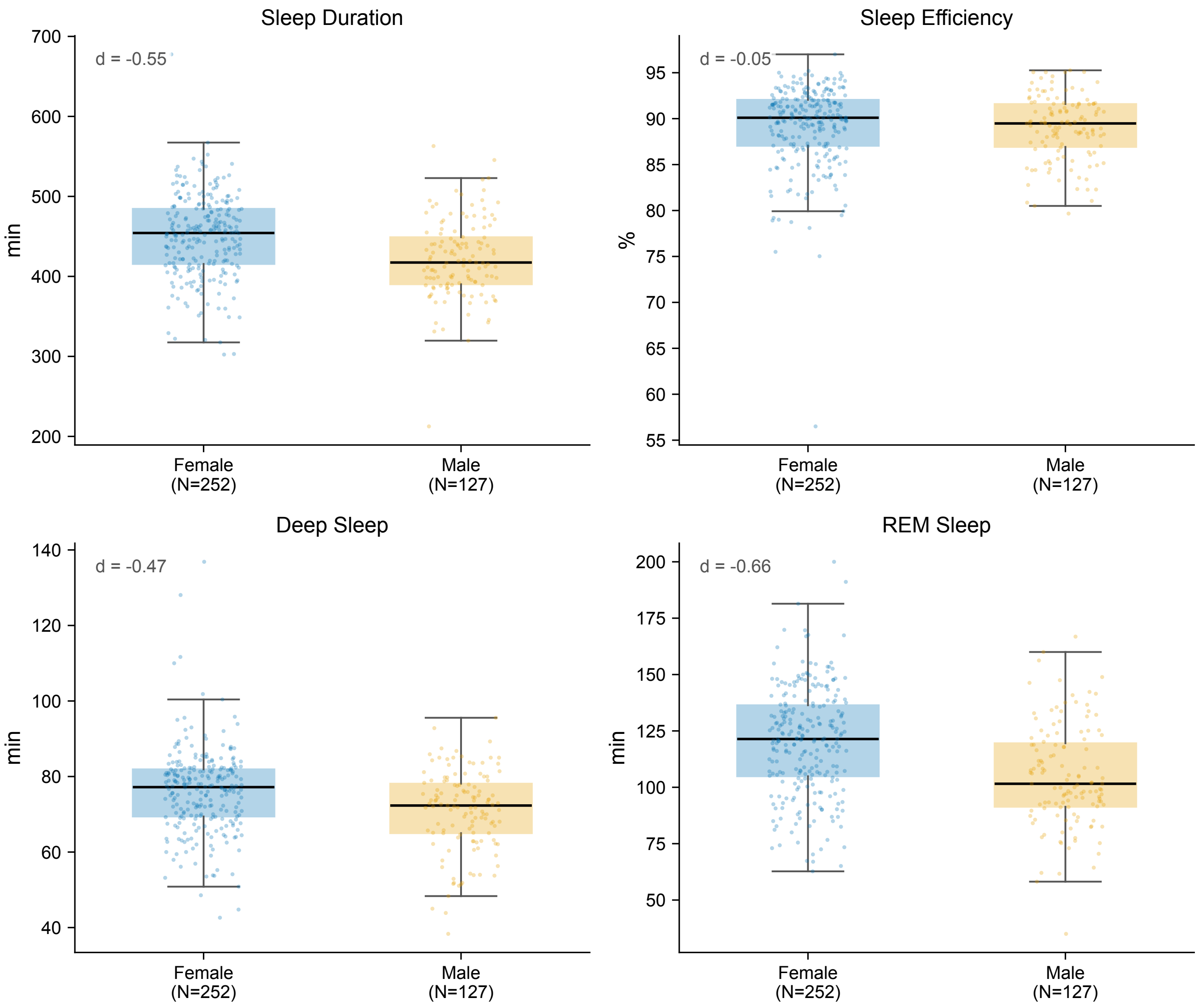

Figure S2. Sleep metrics by gender. Box plots show distributions of sleep duration, sleep efficiency, deep sleep duration, and REM sleep duration for the Female ($N = 252$) and Male ($N = 127$) participants in the analysis cohort with valid sleep-stage data (of 289 and 144 respectively; the remainder lacked scored sleep nights in the data snapshot). Cohen's d for the Male minus Female contrast is annotated on each panel: sleep duration $d = -0.55$, deep sleep $d = -0.47$, REM sleep $d = -0.66$ (all $p < 10^{-4}$); sleep efficiency $d = -0.05$ (not significant). The 9 participants of other gender are omitted owing to small N.

REFERENCES

Dhawale, N., Gandhi, D., Shanmugam, A., Reddy, A., Kubis, H.-P., Driller, M., et al. (2026). Sleep consistency is a low-cost reliable indicator of nocturnal glycemic control. medRxiv preprint. doi:10.64898/2026.03.04.26347496. 227,860 nights, 5,849 adults

Driller, M., Bodner, M. E., Fenuta, A., Stevenson, S., and Suppiah, H. (2026). How does the Sleep Regularity Questionnaire relate to wearable- and diary-derived sleep regularity? *J. Circadian Rhythms* doi:10.5334/jcr.265

Gupta, K., Pirrie, A., Shanmugam, A., Davies, K., Hendi, A., Milnes, R., et al. (2025). Listening in: Introducing the Ultrahuman Cycle Tracking & Ovulation Pro (C&O Pro) feature powered by OvuSense technology. Ultrahuman Science whitepaper. Accessed 29 May 2026. Sensor equivalence Table 1: Ring AIR skin temperature vs OvuSense SWS (CE Class IIa); bias -0.114 °C, MAE 0.046 °C.

Jackson, A. S., Sui, X., Hébert, J. R., Church, T. S., and Blair, S. N. (2009). Role of lifestyle and aging on the longitudinal change in cardiorespiratory fitness. *Arch. Intern. Med.* 169, 1781–1787. doi:10.1001/archinternmed.2009.312

Krishnan, N., Nagesh, R., Singh, P., Asudani, V., Singhal, V., Srinivasan, B., et al. (2024). Sleep heart rate sensing by Ultrahuman Ring AIR demonstrates high overlap with FDA-approved device and consumer-grade wearable. Ultrahuman Science whitepaper. Accessed 28 May 2026

Plews, D. J., Laursen, P. B., Stanley, J., Kilding, A. E., and Buchheit, M. (2013). Training adaptation and heart rate variability in elite endurance athletes: opening the door to effective monitoring. *Sports Med.* 43, 773–781. doi:10.1007/s40279-013-0071-8

Shanmugam, A., Prabhakaran, P., Asudani, V., Joshi, A., Suraj, A., Prasath, A., et al. (2025). Comparison of Ultrahuman Ring AIR temperature sensing at device and hand level reveals superior overlap with gold standards. Ultrahuman Science whitepaper. Accessed 28 May 2026

Tanaka, H., Monahan, K. D., and Seals, D. R. (2001). Age-predicted maximal heart rate revisited. *J. Am. Coll. Cardiol.* 37, 153–156. doi:10.1016/S0735-1097(00)01054-8
